# Immunization with an ApoB-100 Related Peptide Vaccine Attenuates Angiotensin-II Induced Hypertension and Renal Fibrosis in Mice

**DOI:** 10.1371/journal.pone.0131731

**Published:** 2015-06-29

**Authors:** Tomoyuki Honjo, Kuang-Yuh Chyu, Paul C. Dimayuga, Wai Man Lio, Juliana Yano, Portia Trinidad, Xiaoning Zhao, Jianchang Zhou, Bojan Cercek, Prediman K. Shah

**Affiliations:** Oppenheimer Atherosclerosis Research Center, Division of Cardiology, Cedars-Sinai Heart Institute, Cedars-Sinai Medical Center, Los Angeles, California, United States of America; University of Utah School of Medicine, UNITED STATES

## Abstract

Recent studies suggest the potential involvement of CD8+ T cells in the pathogenesis of murine hypertension. We recently reported that immunization with apoB-100 related peptide, p210, modified CD8+ T cell function in angiotensin II (AngII)-infused apoE (-/-) mice. In this study, we hypothesized that p210 vaccine modulates blood pressure in AngII-infused apoE (-/-) mice. Male apoE (-/-) mice were immunized with p210 vaccine and compared to unimmunized controls. At 10 weeks of age, mice were subcutaneously implanted with an osmotic pump which released AngII for 4 weeks. At 13 weeks of age, p210 immunized mice showed significantly lower blood pressure response to AngII compared to controls. CD8+ T cells from p210 immunized mice displayed a different phenotype compared to CD8+ T cells from unimmunized controls. Serum creatinine and urine albumin to creatinine ratio were significantly decreased in p210 immunized mice suggesting that p210 vaccine had renal protective effect. At euthanasia, inflammatory genes IL-6, TNF-α, and MCP-1 in renal tissue were down-regulated by p210 vaccine. Renal fibrosis and pro-fibrotic gene expression were also significantly reduced in p210 immunized mice. To assess the role of CD8+ T cells in these beneficial effects of p210 vaccine, CD8+ T cells were depleted by CD8 depleting antibody in p210 immunized mice. p210 immunized mice with CD8+ T cell depletion developed higher blood pressure compared to mice receiving isotype control. Depletion of CD8+ T cells also increased renal fibrotic gene expression compared to controls. We conclude that immunization with p210 vaccine attenuated AngII-induced hypertension and renal fibrosis. CD8+ T cells modulated by p210 vaccine could play an important role in the anti-hypertensive, anti-fibrotic and renal-protective effect of p210 vaccine.

## Introduction

Hypertension is a major health care problem because of its high prevalence and associated risk of death from stroke, ischemic heart disease, aortic aneurysm, and other vascular diseases. Current clinical paradigm implicates neurohormonal pathways, vascular dysfunction and salt-water imbalance as the main contributors and treatment targets for hypertension. Recently, immune cell responses, especially the role of T cell mediated adaptive immunity, and their crucial contribution to the pathogenesis of hypertension have been reported [1, 2]. Angiotensin II (AngII) is an important mediator of hypertension and T cells play an important role in AngII-induced hypertension [3–5]. T cell activation is augmented by AngII via their functional Renin Angiotensin System (RAS) resulting in increased production of pro-inflammatory cytokines [3, 4]. Among the T cell subsets, a recent report identified a role for CD8+ T cells in the pathogenesis of AngII-induced hypertension [6]. In this report, CD8+ T cells that were detected in the kidneys had evidence of oligoclonal selection, associated with endothelial dysfunction and renal sodium retention. Thus, the studies provided compelling evidence linking CD8+ T cell immune responses in AngII-induced renal dysfunction and hypertension.

We have previously demonstrated that immunization with an apoB-100 related 20 amino acid peptide antigen, p210, reduces atherosclerosis in apoE (-/-) mice mediated by CD8+ T cells independent of antibody production [7]. We also recently reported that immunization with the p210 vaccine modified CD8+ T cell function in AngII-infused apoE (-/-) mice [8]. Based on these immune responses to p210 vaccine and the reported participation of CD8+ T cells in hypertension and kidney dysfunction, we hypothesized that p210 vaccine would modulate the hypertensive response in AngII-infused apoE (-/-) mice.

## Materials and Methods

### Preparation of apoB-100 related peptide for immunization

An apoB-100 related peptide p210 (KTTKQ SFDLS VKAQY KKNKH) was used as an immunogen. One hundred micrograms of native p210 peptide (Euro-Diagnostica AB, Sweden) was conjugated to cationic bovine serum albumin (cBSA) as carrier using a method described previously [7]. The conjugated peptide was freshly prepared prior to each immunization with Alum as adjuvant.

### Experimental animal model

Male apoE (-/-) mice (Jackson Laboratories, Bar Harbor, ME) were housed in a pathogen-free animal facility, fed normal chow, and kept on a 12-hour day/night cycle with ad libitum access to food and water. At 10 weeks of age, an osmotic pump (Alzet model 2004, DURECT Corporation, Cupertino, CA) was subcutaneously implanted under anesthesia in the back to continuously infuse AngII (Sigma-Aldrich, St. Louis, MO) at a dose of 1000ng/Kg/min for 4 weeks [9, 10]. Analgesic was injected subcutaneously prior to the pump implantation to alleviate post-procedure pain. Mice were observed post implantation for recovery from anesthesia and pump implantation under a heat lamp. Mice were checked daily for the duration of the experiment. For the immunization study, a separate group of mice were subcutaneously immunized with p210/cBSA/Alum conjugate in the dorsal area at 7 weeks of age, followed by a booster at 10 and 12 weeks of age [7, 8]. Mice receiving cBSA/alum or PBS served as control groups. AngII pump implantation was performed on the mice at 10 weeks of age, as described above. Mice were sacrificed at 14 weeks of age. The method of euthanasia was overdose of inhalational anesthesia followed by pneumothorax to assure death.

### Ethics Statement

This study was carried out in strict accordance with the recommendations in the Guide for the Care and Use of Laboratory Animals. The protocol (Protocol Number IACUC003093) was approved by the Cedars-Sinai Institutional Animal Care and Use Committee (IACUC). All surgical procedures were performed under inhalational isoflurane anesthesia.

### Blood pressure measurement

Blood pressure was measured by CODA noninvasive tail-cuff system (Kent Scientific Corporation, Torrington, CT). All mice were first trained for five consecutive days before commencing blood pressure measurement. Afterward blood pressure was measured twice a week in the morning from 7 to 13 weeks of age.

### Blood and urine sample analysis

Blood and urine samples were collected at euthanasia. Serum creatinine level was measured using Quantichrom Creatinine Assay kit (BioAssay Systems, Hayward, CA) following the manufacturer’s instruction. Urine albumin was evaluated by the Albuwell M ELISA kit (Exocell, Philadelphia, PA) and urine creatinine was measured using the Creatinine Companion (Exocell, Philadelphia, PA).

### Intracellular cytokine staining

The cervical and axillary lymph nodes were collected from mice at euthanasia and the cells treated with Brefeldin-A for 2 hours in 37°C to arrest extra-cellular secretion of cytokines. Cells were then washed and stained with fluorescently labelled CD4 and CD8 antibodies (eBioscience) overnight. The cells were washed the following day, subjected to fixation and permeabilization for intracellular staining, and stained with fluorescently labelled antibodies to IFN-γ IL-10, IL-12, and TNF-α (eBioscience). Cells were washed, fixed in 1% paraformaldehyde in FACS buffer and analyzed on an LSR Fortessa cell sorter (BD Bioscience).

### Real-time RT-PCR

Total RNA was extracted from kidney with TRIzol reagent (Life Technologies, Carlsbad, CA) according to the manufacturer’s instruction. Total RNA was reverse transcribed into first-strand cDNA using SuperScript III First-Strand Synthesis System (Life Technologies). Quantitative real-time PCR was performed using iQ SYBR Green Supermix and iQ5 Real-Time PCR Detection System (Bio-Rad Laboratories, Hercules, CA). Beta-actin was used for housekeeping gene. The data was analyzed by the delta delta CT method and expressed as arbitrary units (AU) relative to a control sample used as calibrator.

### Assessment of renal fibrosis

At euthanasia, kidneys were harvested, fixed in 4% paraformaldehyde, dehydrated, and embedded in paraffin. Paraffin embedded kidney sections were stained with Masson’s trichrome (Sigma-Aldrich, St. Louis, MO) following the manufacturer’s instructions. For analysis of collagen fiber area, sections were observed under 10x magnification and 5 non-overlapping areas were randomly selected. Each area was then captured under 20x magnification and the percent collagen fiber stain in the captured area was measured using Image Pro plus (Media Cybernetics, Rockville, MD). Collagen fibers surrounding blood vessels were omitted due to the significant variation in the occurrence of vessels in the selected areas. The collagen fiber stained areas from the 5 areas were then averaged. The person who performed the measurement was blinded to the groups.

### Measurement of reactive oxygen species

Kidneys were embedded in OCT compound (Tissue-Tek, Torrance, CA) and snap-frozen. The freshly cut renal cryostat sections (10μm) were used for in situ reactive oxygen species (ROS) measurement. Dihydroethidine hydrochloride (DHE) (5μM, Molecular Probes, Grand Island, NY) was topically applied to the sections for 30 min at 37°C to reveal presence of ROS as red fluorescence by fluorescent microscopy (Carl ZEISS, Thornwood, NY). Quantitative analysis was performed using Image J software.

### In vivo depletion of CD8+ T cells

CD8+ T cell depletion was performed as previously described [11]. p210 immunized mice implanted with a subcutaneous osmotic pump to infuse AngII as described above were intraperitoneally injected with 150μg of anti-CD8 antibody (53-6.7; eBioscience, San Diego, CA) or isotype rat IgG control (Sigma-Aldrich) 3 days before the first immunization and then twice a week thereafter until euthanasia. The efficiency of the depletion was assessed by flow cytometry. Immune response to the injected antibody was assessed using ELISA. Briefly, sera from 5 mice per group were pooled and diluted 1:400 based on prior optimization. Capture antigens used were either CD8 Ab or Isotype at a concentration of 20μg/ml. After blocking with 1% BSA for 2 hours, diluted sera were incubated with the capture antigens for 2 hours, washed, and incubated with HRP-conjugated goat anti mouse IgG antibody (1:5000, Thermo Scientific). The ELISA plates were washed and detection chromogen (ABTS, Southern Biotech) was applied until color development and read on a micro-plate reader at 405 nm.

### Statistics

Data are presented as mean ± SEM. Data were analyzed by ANOVA followed by Newman-Keuls multiple comparison test for multiple group comparison or unpaired two-tailed t-test, unless noted otherwise. P<0.05 was considered statistically significant.

## Results

### Infusion of AngII modulated T cell cytokine profile.

**Fig 1:**
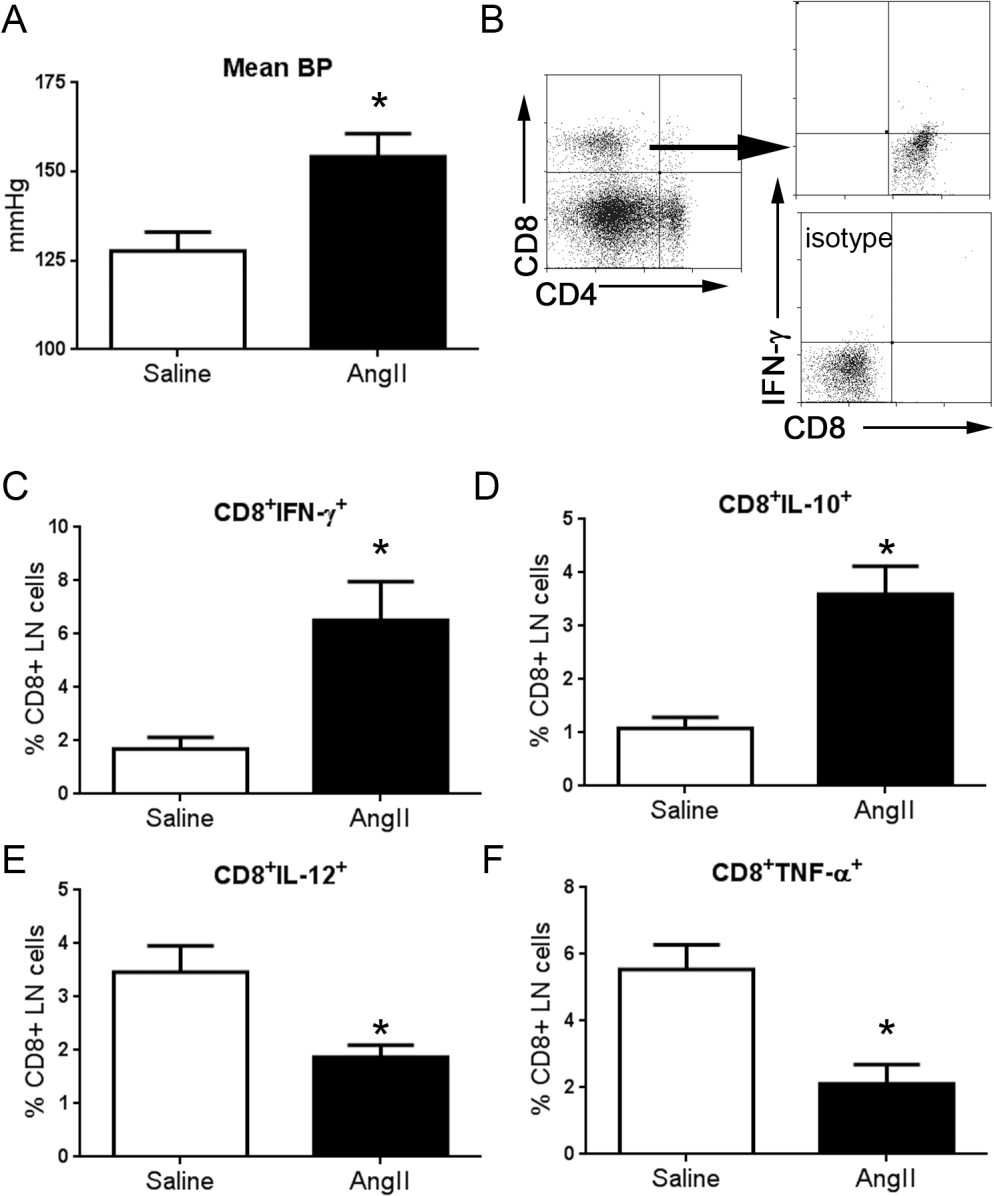
The effect of AngII infusion on blood pressure and CD8+ T cell immune cytokine profile. (A) Mean blood pressure was significantly increased in mice infused with AngII (N = 10) compared to mice infused with saline (N = 8; *P<0.05). (B) Representative scatter plot of gating strategy used for analysis of LN CD8+ T cells in mice infused with saline or AngII. The percentage of CD8+ T cells that were stained with IFN-γ (C), IL-10 (D), IL-12 (E), or TNF-α (F) were plotted on a bar graph. Saline N = 4; AngII N = 5; *P<0.05.

Infusion of AngII into apoE (-/-) mice significantly increased mean blood pressure compared to saline infused mice 3 weeks after infusion pump implantation (154±6 N = 10 vs 128±5 mmHg N = 8, respectively; P<0.01; Fig 1A). The change in blood pressure was associated with significant changes in lymph node (LN) CD8+ T cell cytokine profile (Fig 1B–1F), with significantly increased CD8+IFN-γ+ and CD8+IL-10+ T cells in AngII infused mice. There was also a significant reduction in CD8+IL-12+ and CD8+TNF-α+ T cells in AngII infused mice. LN CD4+IL-10+ T cells were increased, and CD4+TNF-α+ T cells were reduced in the AngII infused mice, with no significant effects on CD4+IFN-γ+ T cells and CD4+IL-12+ T cells (S1 Fig). Thus, AngII infusion altered the cytokine profile of LN T cells.

### Immunization with p210 vaccine attenuated AngII-induced hypertension.

**Fig 2:**
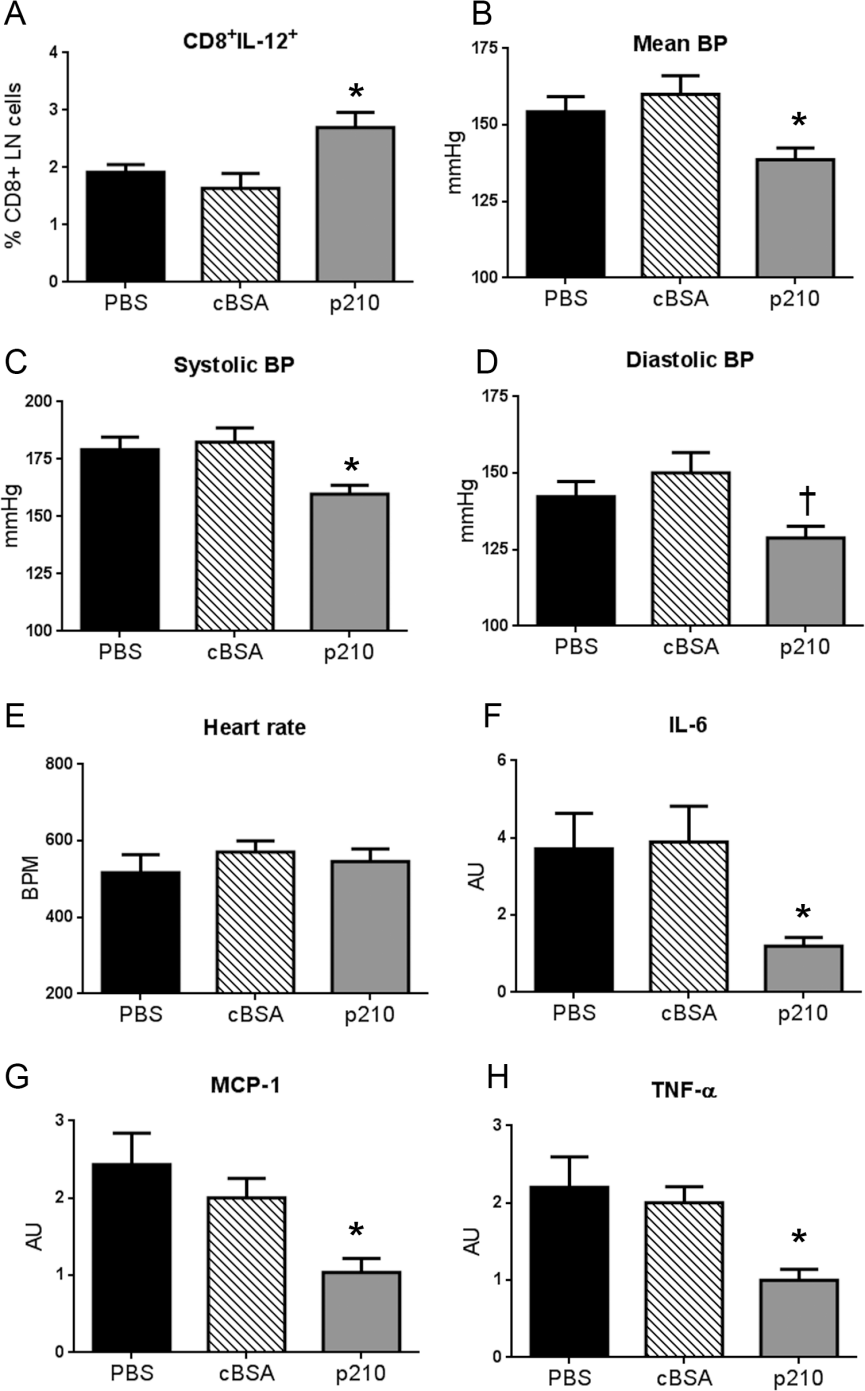
The effect of p210 vaccine on CD8+ T cell profile and hypertension in AngII infused mice. (A) CD8+IL-12+ T cells were significantly increased in LNs of p210 immunized mice (N = 8; P<0.05)) compared to PBS (N = 6) and cBSA (N = 8). p210 immunized mice (N = 9) showed significantly lower mean blood pressure (B) and systolic blood pressure (C) compared to controls (PBS N = 11; cBSA N = 10; *P<0.05). Diastolic blood pressure (D) in p210 mice was significantly reduced compared to cBSA (†P<0.05). There were no differences in heart rate (E) among the groups. Expression of inflammatory genes IL-6 (F), MCP-1 (G), and TNF-α (H) in the kidneys analyzed by real-time RT-PCR were significantly reduced in p210 immunized mice. N of mice: (F) = 8 in each group, (G) = 8–9 in each group, (H) = 10 in each group. *P<0.05 vs. PBS and cBSA.

The p210 vaccine did not result in significant changes in p210-IgG levels (not shown) consistent with our previous findings [7, 8], so further assessment of the immune response was performed on T cells. Immunization with p210 vaccine significantly increased CD8+IL-12+ T cells in the LNs of p210 immunized mice compared to controls (Fig 2A), with no difference in IFN-γ, IL-10, and TNF-α positive CD8+ T cells (data not shown).

Immunization with p210 vaccine per se did not affect blood pressure evidenced by the lack of differences in mean blood pressure after the second immunization injection at 10 weeks of age, before treatment with AngII among the 3 groups (PBS = 120±3 mmHg, N = 11; cBSA = 117±4 mmHg, N = 11; p210 = 118±4 mmHg, N = 10). At 13 weeks of age, 3 weeks after implantation of the AngII osmotic infusion pump, mean blood pressure was significantly lower in p210 immunized mice compared to PBS and cBSA control groups (Fig 2B). There was significantly reduced systolic and diastolic blood pressure in the p210 immunized mice, and no significant difference in heart rate (Fig 2C–2E).

### p210 vaccination decreased AngII-induced renal inflammatory gene expression.

AngII induced renal inflammation and hypertension is mediated by interleukin 6 (IL-6), and characterized by increased expression of tumor necrosis factor (TNF)-α, and monocyte chemoattractant protein-1 (MCP-1) [3, 9, 12–14]. We therefore examined the effect of p210 immunization on the expression of these inflammatory genes in the kidney. Renal IL-6, TNF-α, and MCP-1 expression were significantly down-regulated in p210 immunized mice compared to the control groups (Fig 2F–2H).

### p210 vaccination protected against renal dysfunction.

To explore further the renal effects of the p210 vaccine, we assessed renal function by measuring serum creatinine (Cr.) and urine albumin to creatinine ratio (ACR). p210 immunized mice showed significantly lower serum Cr. and urine ACR compared to controls indicating that p210 vaccine attenuated AngII-induce renal dysfunction (Table 1).

**Table 1:** Serum creatinine and urine albumin to creatine ratio at euthanasia.

	PBS	cBSA	p210
Serum Cr. (mg/dL)	1.33 ± 0.1	1.08 ± 0.2	0.57 ± 0.1*
Urine ACR (μg/mg)	223 ± 30	191 ± 34	99 ± 12*

N of mice for serum Cr.: PBS = 9, cBSA = 11, p210 = 12. N of mice for urine ACR: PBS = 15, cBSA = 11, p210 = 11.

*p < 0.05 vs. PBS and cBSA.

### p210 vaccination decreased ROS generation and NOX1 expression.

**Fig 3:**
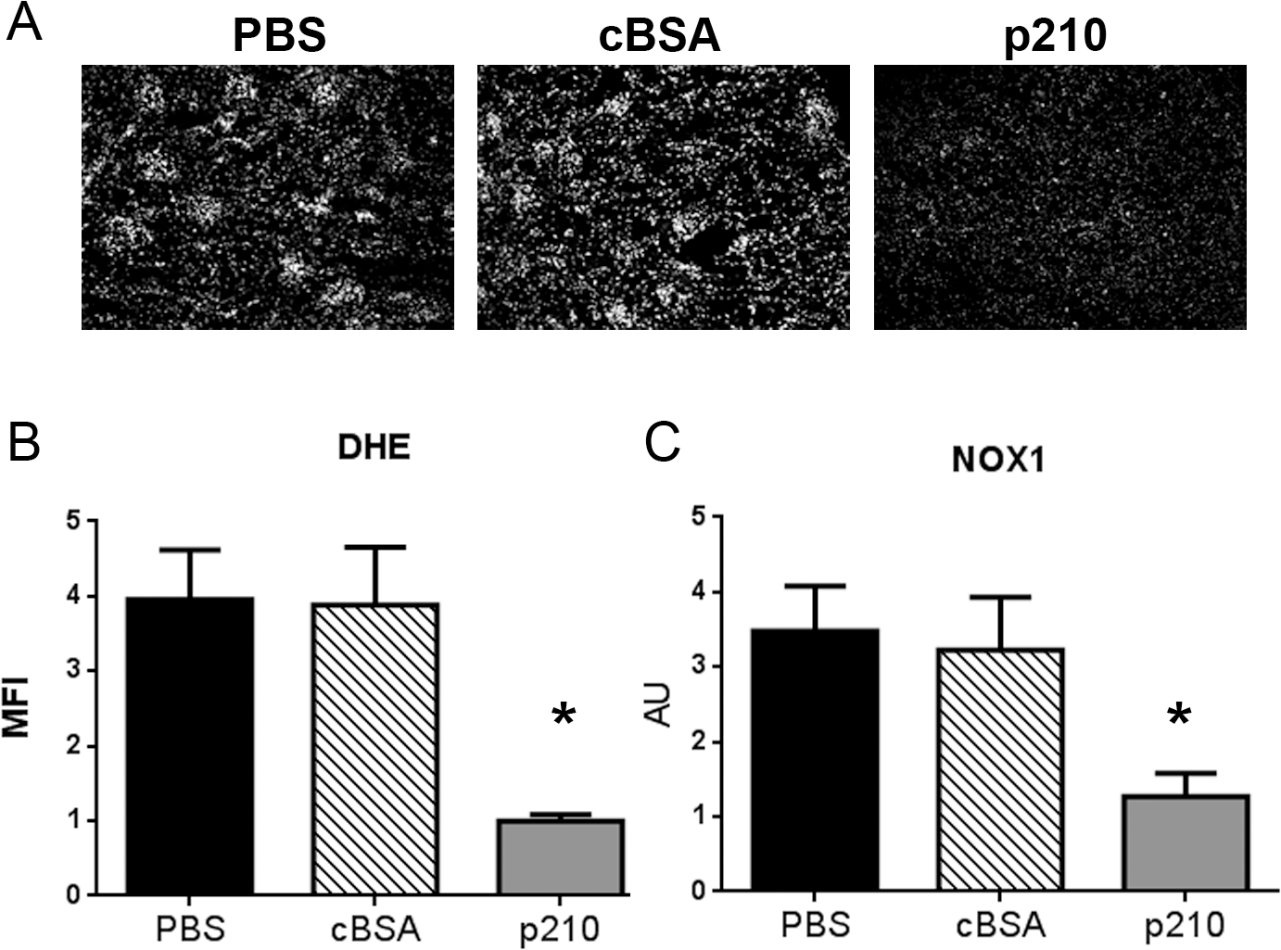
p210 vaccine attenuated AngII-induced superoxide production in kidney. (A) Representative pictures of in situ DHE staining at 10x magnification. (B) Densitometric analysis of DHE fluorescence. Immunization with p210 vaccine significantly reduced AngII-induced renal ROS production. N = 4 in each group. (C) The mRNA expression of NOX1 in kidney was analyzed by quantitative real-time RT-PCR. Immunization with p210 vaccine significantly down-regulated renal NOX1 expression. N of mice = 8 in each group. *P<0.05 vs. PBS and cBSA.

Reactive oxygen species (ROS) generation has been implicated in the pathogenesis of AngII-induced hypertension, mediated by the NADPH oxidase genes. We therefore tested ROS generation and NADPH oxidase gene expression in the kidneys of the immunized mice compared to controls. In situ dihydroethidine hydrochloride (DHE) staining showed that glomerular reactive oxygen species (ROS) production was reduced in p210 immunized mice (Fig 3A) as quantified by mean fluorescent intensity (Fig 3B). p210 immunized mice also had reduced NOX1 expression in kidneys (Fig 3C). There was a significant reduction in the expression of p22phox and p47phox in the cBSA group, but not in the p210 group (S2 Fig) suggesting non-specific or non-relevant effects given that there were no other beneficial effects observed in the cBSA group. The expression of other NADPH oxidase genes examined (p67phox, NOX2, and NOX4; S2 Fig) did not differ among the groups.

### p210 vaccination reduced renal fibrosis and pro-fibrotic gene expression.

**Fig 4:**
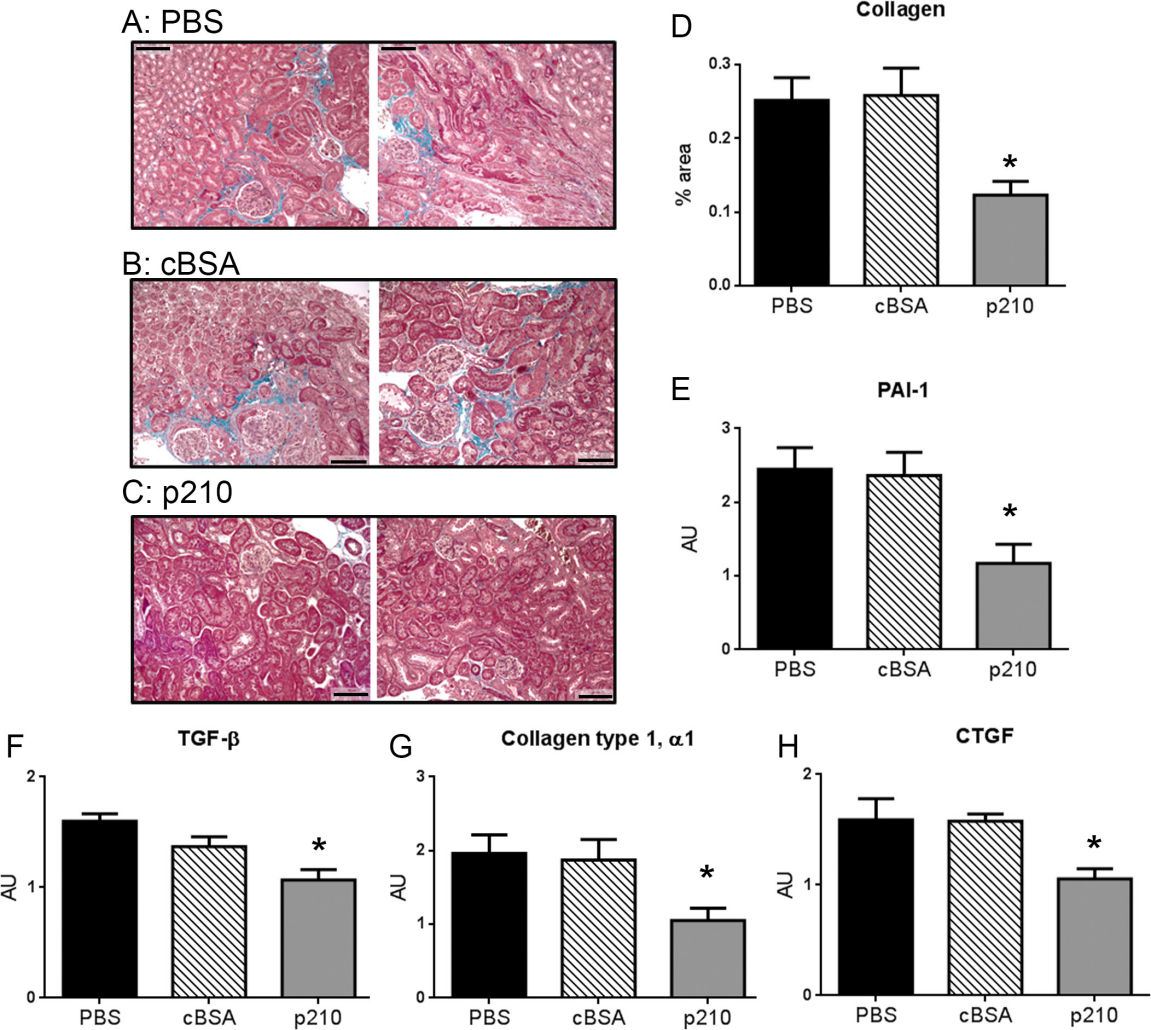
p210 vaccine attenuated AngII-induced renal fibrosis. (A-C) Representative pictures of Masson Trichrome staining of the kidney. Blue staining indicates renal fibrosis. A: PBS, B: cBSA, C: p210. 20x magnification; Bar = 0.1mm. (D) Quantitative analysis of positive area. Immunization with p210 vaccine significantly attenuated AngII-induced renal fibrosis. N of mice: PBS = 11, cBSA = 10, p210 = 6. *P<0.05 vs. p210. The mRNA expression of (E) PAI-1, (F) TGF-β, (G) Collagen type I, α1, and (H) CTGF in kidney were analyzed by quantitative real-time RT-PCR. Immunization with p210 vaccine significantly decreased renal pro-fibrotic gene expression. N of mice: (E) = 4 in each group, (F) = 9–10 in each group, (G) = 9–10 in each group, (H) = 9 in each group. *P<0.05 vs. PBS and cBSA.

To determine the effects of p210 vaccine on renal fibrosis, Masson Trichrome staining (Fig 4A–4C) and renal pro-fibrotic gene expression analysis were performed. Quantification of collagen-stained area showed that p210 immunized mice had significantly reduced renal fibrosis compared to PBS and cBSA controls (0.12±0.02%, N = 6 vs. 0.25±0.03%, N = 11 and 0.26±0.04%, N = 10, respectively; Fig 4D). Quantitative real-time RT-PCR demonstrated that renal expression of pro-fibrotic genes plasminogen activator inhibitor 1 (PAI-1, Fig 4E), transforming growth factor (TGF)-β (Fig 4F), collagen type I, α-1 (Fig 4G), and connective tissue growth factor (CTGF, Fig 4H) were significantly down-regulated in p210 immunized mice.

### Depletion of CD8+ T cells reversed the anti-hypertensive effect of p210 vaccine.

**Fig 5:**
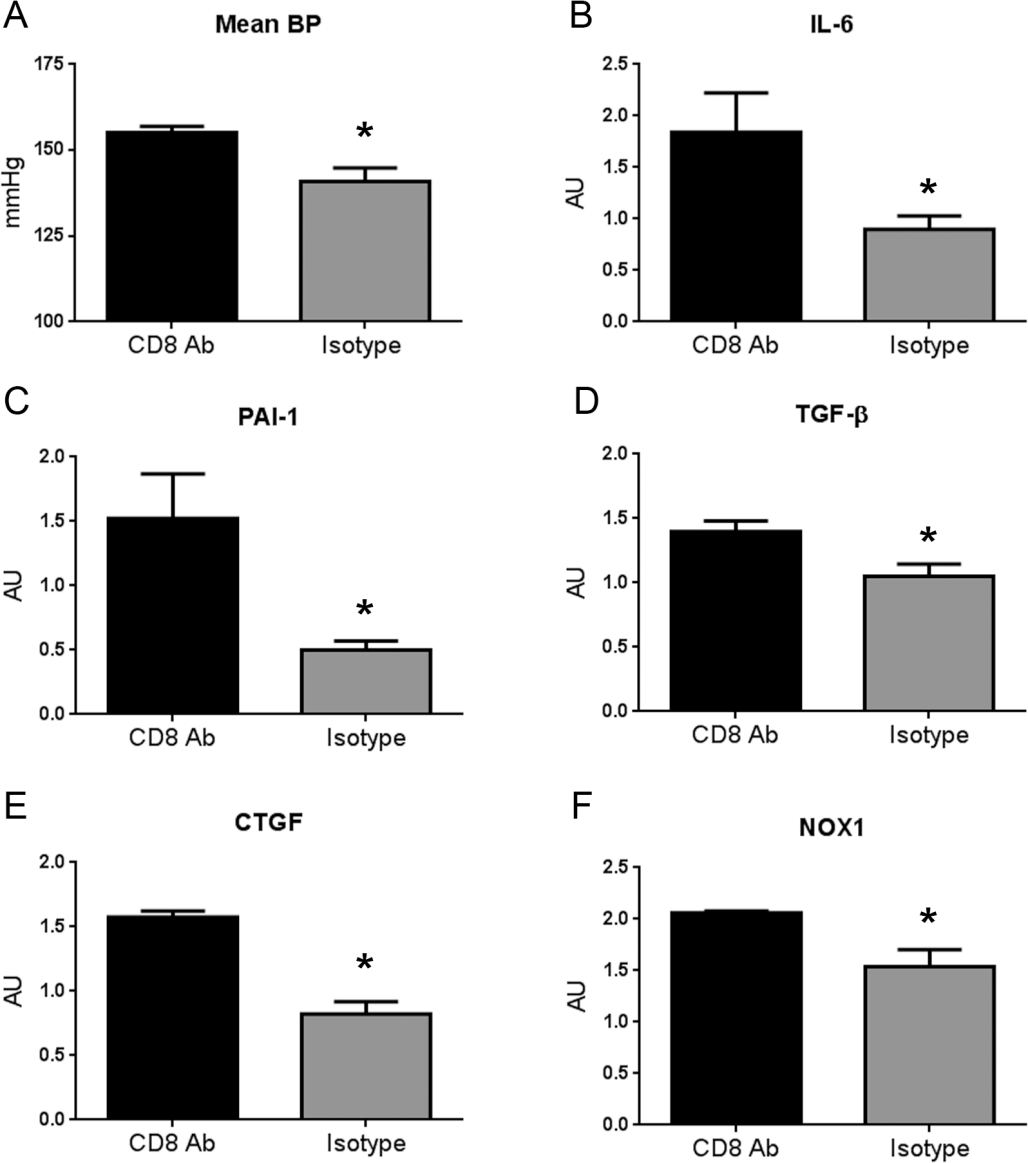
p210 immunized mice with CD8+ T cell depletion developed higher blood pressure. (A) CD8 antibody (CD8 Ab) group showed significantly higher mean blood pressure compared to Isotype group at 13 weeks of age (CD8 Ab = 155±2 mmHg, N = 6; Isotype = 141±4 mmHg, N = 9; *P<0.05). Gene expression of inflammatory cytokine IL-6 (B), pro-fibrotic genes PAI-1 (C), TGF-β (D) and CTGF (E), and NADPH oxidase gene NOX1 (F) were all significantly increased in CD8 Ab group compared to Isotype. N = 4–5 each; *P<0.05 vs CD8 Ab.

Our previous report showed that the athero-protective effect of the vaccine was mediated by p210 modulated CD8+ T cells [7]. Recently, we also demonstrated that p210 vaccine protected against AngII-induced aortic aneurysm formation through modification of CD8+ T cells [8]. Given that the p210 vaccine provoked a CD8+ T cell response, we determined if the anti-hypertensive effect of p210 vaccine was also mediated by CD8+ T cells by treating p210 immunized mice with a CD8 depleting antibody. p210 immunized mice with CD8+ T cell depletion developed higher mean blood pressure response to AngII compared to similarly treated mice receiving isotype control antibody supporting the role of CD8+ T cells in the anti-hypertensive effect of p210 vaccination (Fig 5A). To further investigate the involvement of CD8+ T cells in the protective effect of p210 immunization, renal cytokine and chemokine gene expression were assessed. There was significantly increased IL-6 expression after CD8 depletion in p210 immunized mice compared to the isotype control (Fig 5B). There were no significant differences in renal MCP-1 and TNF-α expression (not shown).

PAI-1, TGF-β, CTGF, and NOX1 mRNA expression were also significantly higher after CD8 depletion compared to isotype control (Fig 5C–5F, respectively), with no significant difference in Collagen type 1, α1 expression (not shown). There was no significant difference in renal collagen stain (S3 Fig)

## Discussion

In this study, we report that infusion of AngII into apoE (-/-) mice modified T cell cytokine profile and induced hypertension. We further show that the apoB-100 related peptide p210 vaccine attenuated AngII-induced hypertension and renal dysfunction. The reno-protective effect of p210 vaccine was associated with reduced renal inflammation and fibrosis. By using CD8 depleting antibody in vivo, we demonstrated that p210 modulated CD8+ T cells played a crucial role in the anti-hypertensive effects of p210 vaccine.

We conceived of the approach to vaccinate mice against AngII-induced hypertension after completing our studies that showed favorable effects of p210 immunization in atherosclerosis and AngII-induced aortic aneurysm formation and rupture. In these studies, we found that the p210 vaccine modulated CD8+ T cells and their function. Given the recent evidence of CD8+ T cell involvement in hypertension pathophysiology [6], we therefore tested the vaccine on AngII-induced hypertension with the expectation that modulation of CD8+ T cells would alter the hypertensive response to AngII.

The potential involvement of the immune system in the pathogenesis of hypertension has been known for more than 40 years [1, 2, 15]. Only recently, with the advancement of immunological knowledge and tools, has convincing evidence accumulated on the involvement of T cells in hypertension through the effect on kidneys [2]. A recent report by Trott et al demonstrated the essential role of CD8+ T cells in mediating Ang-II induced hypertension and described clonality in CD8+ T cells in the kidneys of mice infused with AngII [6]. The pro-hypertension role of CD8+ T cells was demonstrated in a CD8 deficient mouse model and adoptive transfer of T cells into immune-deficient Rag-1-/- mice. The report supports our hypothesis that modulation of CD8+ T cell function may be a viable target to control hypertension in the AngII infusion model.

AngII stimulates inflammatory responses and regulates renal cell growth and extracellular matrix synthesis by multiple fibrotic pathways [16]. In our study, we demonstrated that immunization with p210 vaccine significantly reduced IL-6, MCP-1, and TNF-α expression in the kidney. AngII-induced hypertension is significantly attenuated in IL-6 deficient mice, thus providing evidence that IL-6 is a potent mediator of hypertension in this model [9]. AngII induced pro-fibrotic gene expression in the kidney was reduced in IL-6 deficient mice [9]. MCP-1 enhanced IL-6 activity and inflammation through the reported IL-6/MCP-1 amplification loop [17]. TNF-α is a well-known inflammatory cytokine and appears to have an important role in hypertension and renal fibrosis [3, 13, 14]. Taken altogether, the reduction of these inflammatory cytokines in the kidney by p210 vaccine may contribute to the anti-hypertensive effect of p210 vaccine.

ROS generation mediated by NOX1 is another pathway involved in AngII induced hypertension [18]. Reduced ROS generation in the kidneys of the p210 immunized mice associated with reduced renal NOX1 expression suggests that ROS generation mediated by NOX1 is also favorably affected by the p210 vaccine.

AngII also increases renal expression of pro-fibrotic genes such as TGF-β, CTGF, PAI-1, and type I collagen [16, 19]. Our results show that immunization with p210 vaccine significantly attenuated AngII-induced renal fibrosis with down-regulation of renal TGF-β, CTGF, PAI-1, and type I collagen gene expression. Thus, in addition to the anti-hypertensive effect of p210 vaccine, reduced renal fibrosis was also observed supporting a role for renal-protective effects of the vaccine. The pro-inflammatory and pro-fibrotic responses in AngII-induced hypertension are likely not exclusive of each other given the reported interaction and feed-forward mechanisms of the two pathways, specifically of IL-6 and TGF-β [20].

To provide evidence for the role of CD8+ T cells in the protective effect of the p210 vaccine, we performed CD8 depletion experiments. Depletion of CD8+ T cells resulted in the reversal of the anti-hypertensive effect of the vaccine and increased renal IL-6, but not MCP-1 and TNF-α. The results suggest that the CD8+ T cell response provoked by p210 immunization does not directly affect MCP-1 and TNF-α expression. Although MCP-1 and IL-6 are involved in a signaling amplification loop [17], evidence suggests that MCP-1 is not directly involved in the hypertensive response to AngII [21]. TNF-α on the other hand has been shown to be involved in the hypertensive response, but a study by Sriramula et al provided evidence that the role of TNF-α in hypertension is likely localized to the brain RAS by using intracerebroventricular (ICV) infusion of the TNF-α inhibitor etanercept in AngII treated mice [22]. AngII induced hypertension was reduced in ICV etanercept infused mice, with reduced pro-inflammatory cytokine expression and increased IL-10 levels, providing intriguing evidence for the role of the neurohormonal/inflammatory axis in hypertension [22].

The increased expression of IL-6 after CD8+ T cell depletion in immunized mice was associated with increased TGF-β expression. The role of IL-6/TGF-β interaction has been reported previously, specifically in fibrosis attributed to TGF-β signaling [23]. More relevant to our study, IL-6 was reported to be essential to TGF-β expression during cardiac fibrosis induced by AngII [20]. Interestingly, the same investigators recently reported that CD8+ T cells are required for AngII mediated cardiac inflammation [24]. These reports are broadly consistent with our results.

The recent report confirming the pathophysiologic role of CD8+ T cells in hypertension [6] is consistent with our approach to modulate CD8+ T cell immune response to control hypertension. In our study, infusion of apoE (-/-) mice with AngII significantly changed the cytokine profile of CD8+ T cells, supporting the role of this T cell subtype in hypertension. Immunization with p210 also modified CD8+ T cell cytokine profile, but in such a way that the pathogenic effect induced by AngII infusion was reduced. This is supported by the negation of the protective effect of p210 immunization when CD8+ T cells were depleted. Thus, although CD8+ T cells may promote the hypertensive response to AngII, p210 vaccination provoked a response that was able to alter this to a more protective role.

The effect of the depletion experiment may seem at odds with the reported role of CD8+ T cells in mediating AngII-induced hypertension [6]. In the report, absence of CD8+ T cells in CD8-/- mice resulted in reduced hypertension in response to AngII. This was further verified in the same report by adoptive transfer of CD8+ T cells into immune-deficient Rag-1-/- mice. Thus, one would expect that depletion of CD8+ T cells with the CD8 Ab in our studies result in reduced hypertensive response. However, unlike complete absence of the cell types in the knockout mice used by Trott et al, antibody-mediated depletion rarely results in complete elimination of the target cell, especially if performed over a period of time where the host may mount an immune response against the injected antibody, as shown in S3 Fig, resulting in reduced potency of the depletion as we have previously reported [8]. In our previous report, a similar antibody depletion experiment resulted in about 60% depletion of CD8+ T cells [8]. Thus, it appears that the remaining CD8+ T cells in the current study were able to mount a response to the AngII challenge and maintain it.

An interesting outcome of the depletion experiment is the apparent lack of effect on vaccine-mediated reduction of renal fibrosis. This result suggests that AngII-induced renal fibrosis is more sensitive to perturbations of CD8+ T cell counts. There is possibly a threshold of CD8+ T cell count in the fibrotic response such that no observable effect (or a delayed effect) will occur below that threshold. This concept remains to be tested.

It is still unclear how the kidney becomes an organ involved in immune signaling. However, previous reports provide some insight into the process. In a rat model of five-sixths nephrectomy, renal dendritic cells generated and presented antigenic peptides from albumin that activated syngeneic CD8+ T cells, providing proof of an immune response to renal self-antigens [25]. In another report using transgenic mice selectively expressing ovalbumin as antigen in glomerular podocytes, repetitive co-injection of OT-I and OT-II cells that are reactive to ovalbumin in the context of MHC-I and MHC-II, respectively, resulted in significant tubulointerstitium inflammation and damage [26]. Depletion of dendritic cells attenuated the renal immunopathology in the study [26], providing additional evidence that the immune activation and response to renal antigens is a crucial mediator of renal disease. It is noteworthy that the kidney can express apoB-100 [27] making it a potential source of self-antigens given that the antigen used in our study, p210, is a peptide from apoB-100. Although possibly non-immunogenic in their native forms, modification during inflammatory processes may alter these self-proteins to become potentially immunogenic and thus provoke immune responses. One of these potential modifications includes the formation of isoketals, recently shown to activate T cells and promote hypertension [28].

Our study has limitations inherent to the model used, and therefore needs to be replicated in other models of hypertension. However, perturbations in CD8+ T cells have been reported in human hypertensive patients [29] supporting the notion that CD8+ T cell modulation may be a viable target in controlling hypertension. Our study further provides a vaccination strategy against hypertension with a specific peptide antigen, and the anti-hypertensive effects are mediated by CD8+ T cells. We conclude that CD8+ T cell modulation in p210 immunized mice blunted the AngII-induced hypertension and renal dysfunction, and reduced inflammatory and fibrotic gene expression.

## Supporting Information

S1 Fig Effect of AngII on CD4+ T cell cytokine profile. Infusion of apoE (-/-) mice with AngII had no significant effect on CD4+IFN-γ+ T cells in LNs (A) but significantly increased CD4+IL-10+ T cells (B). CD4+IL-12+ T cells (C) were similar between saline control and AngII-infused mice, but CD4+TNF-α+ T cells were significantly reduced in AngII infused mice (D). Saline N = 4; AngII N = 5; *P<0.05.
(PDF)

S2 Fig Effect of p210 immunization on renal NADPH oxidase gene expression. There were no significant differences in NOX2 (A) and NOX4 (B) mRNA expression among the groups. Both p22phox (C) and p47phox (D) mRNA expression were reduced in the cBSA control group compared to the PBS control group and p210 group. The mRNA expression for p67phox (E) was also trending lower in the cBSA control group but was not statistically significant. *P<0.05 vs. PBS and p210.
(PDF)

S3 Fig Renal collagen stain area in CD8-depleted mice. (B) Renal collagen stained area in p210 vaccinated mice treated with CD8 Ab (N = 7) or Isotype (N = 4) injections.
(PDF)

S4 Fig Effect of CD8 Ab injections on serum IgG against rat IgG. Presence of mouse IgG against injected rat IgG in pooled serum of p210 vaccinated mice treated with CD8 antibody (CD8 Ab) or Isotype injections. Capture antigen used was CD8 Ab (A) or Isotype IgG (B). Control is p210 vaccinated mice injected with saline. Serum was pooled from 5 mice per group.
(PDF)
