# Supplementary figures and images for: Immunization with an ApoB-100 Related Peptide Vaccine Attenuates Angiotensin-II Induced Hypertension and Renal Fibrosis in Mice
Source: PLoS One. 2015 Jun 29;10(6):e0131731. doi: 10.1371/journal.pone.0131731 (PMC4486456; doi:10.1371/journal.pone.0131731)

**A****CD4+IFN+**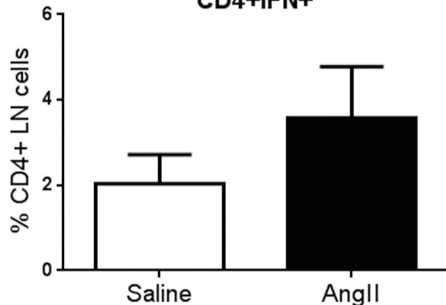**B****CD4+IL-10+**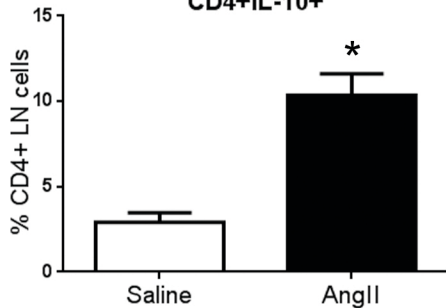**C****CD4+IL-12+**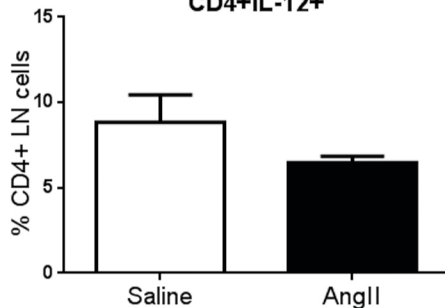**D****CD4+TNF+**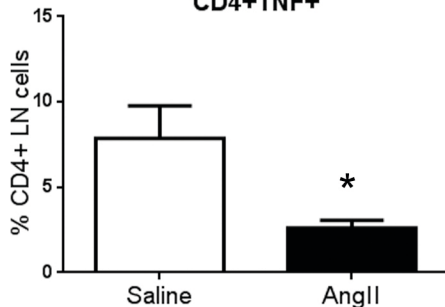

**S1 Figure: Effect of AngII on CD4+ T cell cytokine profile.**

Supplement: S1 Fig — Infusion of apoE (-/-) mice with AngII had no significant effect on CD4+IFN-γ+ T cells in LNs (A) but significantly increased CD4+IL-10+ T cells (B). CD4+IL-12+ T cells (C) were similar between saline control and AngII-infused mice, but CD4+TNF-α+ T cells were significantly reduced in AngII infused mice (D). Saline N = 4; AngII N = 5; *P<0.05. (PDF) [file pone.0131731.s001.pdf]

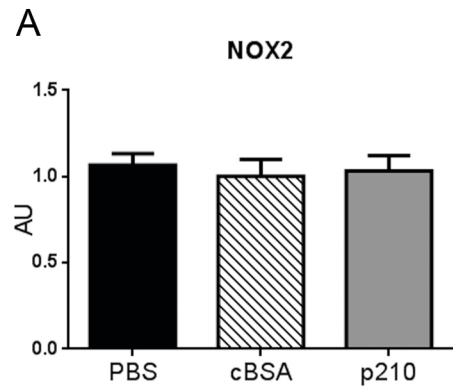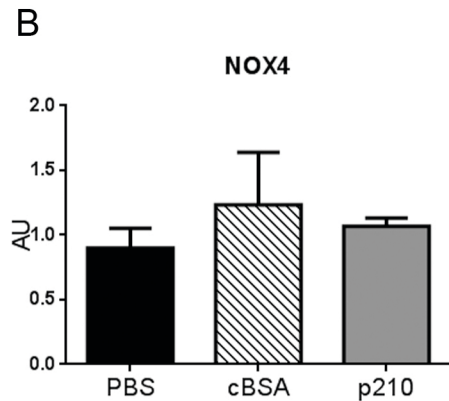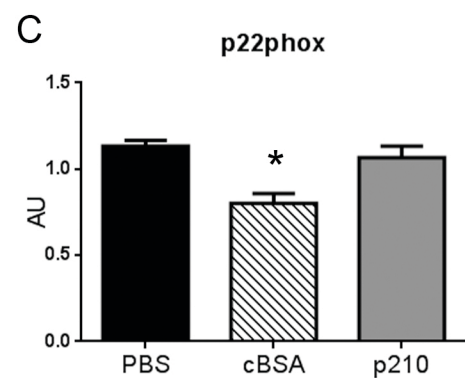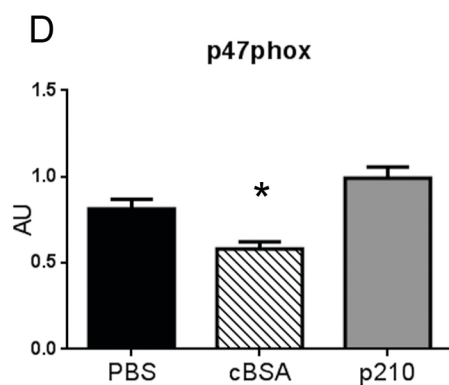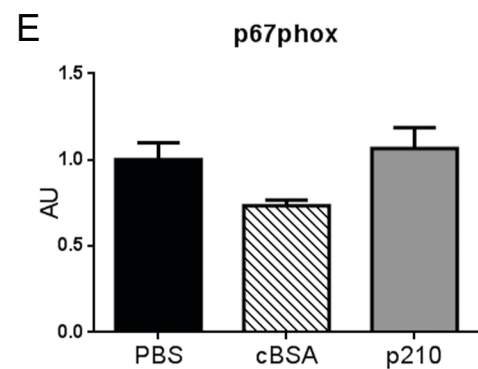

**S2 Figure: Effect of p210 immunization on renal NADPH oxidase gene expression.**

Supplement: S2 Fig — There were no significant differences in NOX2 (A) and NOX4 (B) mRNA expression among the groups. Both p22phox (C) and p47phox (D) mRNA expression were reduced in the cBSA control group compared to the PBS control group and p210 group. The mRNA expression for p67phox (E) was also trending lower in the cBSA control group but was not statistically significant. *P<0.05 vs. PBS and p210. (PDF) [file pone.0131731.s002.pdf]

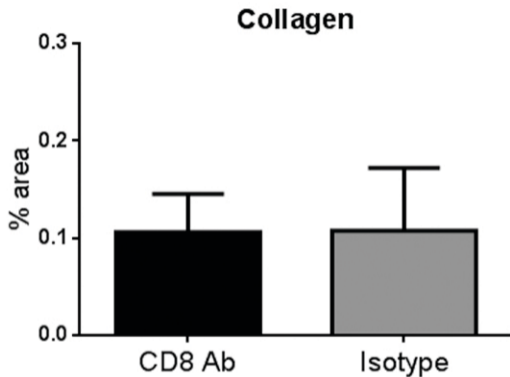

**S3 Figure: Renal collagen stain area in CD8-depleted mice.**

Supplement: S3 Fig — (B) Renal collagen stained area in p210 vaccinated mice treated with CD8 Ab (N = 7) or Isotype (N = 4) injections. (PDF) [file pone.0131731.s003.pdf]

A

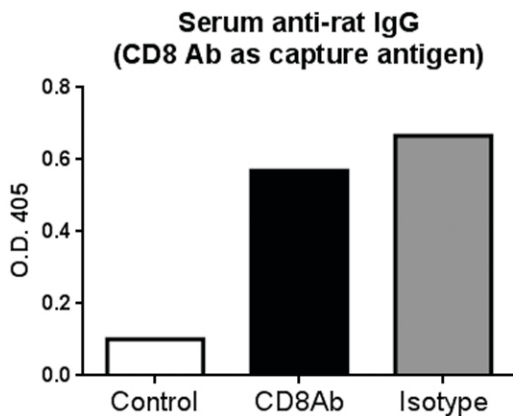

B

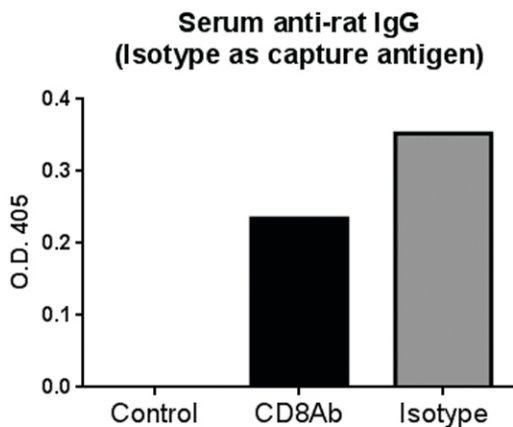

**S4 Figure: Effect of CD8 Ab injections on serum IgG against rat IgG.**

Supplement: S4 Fig — Presence of mouse IgG against injected rat IgG in pooled serum of p210 vaccinated mice treated with CD8 antibody (CD8 Ab) or Isotype injections. Capture antigen used was CD8 Ab (A) or Isotype IgG (B). Control is p210 vaccinated mice injected with saline. Serum was pooled from 5 mice per group. (PDF) [file pone.0131731.s004.pdf]
